# Candidate gene association study in pediatric acute lymphoblastic leukemia evaluated by Bayesian network based Bayesian multilevel analysis of relevance

**DOI:** 10.1186/1755-8794-5-42

**Published:** 2012-09-28

**Authors:** Orsolya Lautner-Csorba, András Gézsi, Ágnes F Semsei, Péter Antal, Dániel J Erdélyi, Géza Schermann, Nóra Kutszegi, Katalin Csordás, Márta Hegyi, Gábor Kovács, András Falus, Csaba Szalai

**Affiliations:** 1 Department of Genetics, Cell- and Immunobiology, Semmelweis University, Budapest, Nagyvárad tér 4, H-1089, Hungary; 2 Department of Measurement and Information Systems, University of Technology and Economics, Budapest, Hungary; 3 2nd Department of Pediatrics, Semmelweis University, Budapest, Hungary; 4 Heim Pal Children Hospital, Budapest, Hungary; 5 Csertex Research Laboratory, Budapest, Hungary

**Keywords:** ALL susceptibility, Bayesian network based Bayesian multilevel analysis of relevance (BN-BMLA), Frequentist-based statistical analysis, Gene-gene interaction, Genetics, Genomics, Risk factors, Direct and indirect interactions, Transitive interaction, Strong relevance, Systems biology

## Abstract

**Background:**

We carried out a candidate gene association study in pediatric acute lymphoblastic leukemia (ALL) to identify possible genetic risk factors in a Hungarian population.

**Methods:**

The results were evaluated with traditional statistical methods and with our newly developed Bayesian network based Bayesian multilevel analysis of relevance (BN-BMLA) method. We collected genomic DNA and clinical data from 543 children, who underwent chemotherapy due to ALL, and 529 healthy controls. Altogether 66 single nucleotide polymorphisms (SNPs) in 19 candidate genes were genotyped.

**Results:**

With logistic regression, we identified 6 SNPs in the *ARID5B* and *IKZF1* genes associated with increased risk to B-cell ALL, and two SNPs in the *STAT3* gene, which decreased the risk to hyperdiploid ALL. Because the associated SNPs were in linkage in each gene, these associations corresponded to one signal per gene. The odds ratio (OR) associated with the tag SNPs were: OR = 1.69, P = 2.22x10^-7^ for rs4132601 (*IKZF1*), OR = 1.53, P = 1.95x10^-5^ for rs10821936 (*ARID5B*) and OR = 0.64, P = 2.32x10^-4^ for rs12949918 (*STAT3*). With the BN-BMLA we confirmed the findings of the frequentist-based method and received additional information about the nature of the relations between the SNPs and the disease. E.g. the rs10821936 in *ARID5B* and rs17405722 in *STAT3* showed a weak interaction, and in case of T-cell lineage sample group, the gender showed a weak interaction with three SNPs in three genes. In the hyperdiploid patient group the BN-BMLA detected a strong interaction among SNPs in the *NOTCH1*, *STAT1*, *STAT3* and *BCL2* genes. Evaluating the survival rate of the patients with ALL, the BN-BMLA showed that besides risk groups and subtypes, genetic variations in the *BAX* and *CEBPA* genes might also influence the probability of survival of the patients.

**Conclusions:**

In the present study we confirmed the roles of genetic variations in *ARID5B* and *IKZF1* in the susceptibility to B-cell ALL. With the newly developed BN-BMLA method several gene-gene, gene-phenotype and phenotype-phenotype connections were revealed. We showed several advantageous features of the new method, and suggested that in gene association studies the BN-BMLA might be a useful supplementary to the traditional frequentist-based statistical method.

## Background

Pediatric acute lymphoblastic leukemia (ALL) is a clonal disease of a lymphoblast and the most common malignancy of all childhood cancers. It is generally accepted that tumorogenesis results from complex interplay between inherited genetic background and specific environmental exposure [1]. In the last decade several genome wide and candidate gene association studies have been carried out and revealed a number of genes and genetic variations, which might influence the risk to the disease [2-6]. In gene association studies, due to the high number of inconclusive results, it is generally accepted that the role of a gene or a genetic variation can only be acknowledged, if it is confirmed by independent studies.

In gene association studies it is well-known, that the traditional frequentist-based statistical methods have several limitations, like the difficult handling of the multiple testing problem and model complexity, the inappropriate approach towards complex traits (i.e. disregarding the role of high-number of weak factors, gene-gene interactions, pathway-based interpretation), and the high redundancy of predictors, e.g. the discovery of non-causal, transitively associated descriptors [7,8].

Recently, we have introduced a new statistical methodology, named Bayesian network based Bayesian multilevel analysis of relevance (BN-BMLA), which supports association analysis by estimating posteriors of strong relevance [9-11]. First, we tested the BN-BMLA method in a case–control setup using artificial datasets for identifying interactions and conditional relevance [12]. The BN-BMLA was proven to be superior over other multivariate methods using conditional models designed to detect associations between genotypic variables and the target variable. Later, we also tested the method in a real world dataset of a partial genome association study in asthma [13]. In this latter study we found that next to the directly associated genes identified by the frequentist-based methods (χ^2^ test, multivariate logistic regression and multifactor dimensionality reduction), the BN-BMLA could detect plausible additional genes involved in gene-gene interactions or genes which were indirectly associated with asthma, i.e. showed transitive association via other variables (e.g. via rhinitis).

As could be seen from the results, the advantage was not only that the BN-BMLA could detect more relevant variables, but the Bayesian networks offered a rich language for the detailed representation of types of relevance, including causal, acausal, and multitarget aspects. Additionally, Bayesian statistics offers an automated and normative solution for the multiple hypothesis testing problems. The BN-BMLA extends the scope of local ‘causal’ discovery methods, and because of the direct interpretation of Bayesian posteriors, contrary to p-values from the frequentist-based approach, makes it an ideal candidate for creating probabilistic knowledge bases to support off-line meta-analysis and fusion of background knowledge.

In the present study we use a traditional frequentist-based method (logistic regression) and the BN-BMLA for evaluating the results of a candidate gene association study in ALL. From the scientific literature and databases we selected 66 SNPs in 19 known candidate genes and investigated with the two methods whether the presence of these polymorphisms was associated with ALL in our population, and whether any of these alleles influenced some disease characteristics and the outcome of the therapy in the affected individuals.

## Methods

### Study population and definitions

In a retrospective manner, DNA was obtained from 543 children (mean age at diagnosis: 6.4 ± 4.2 years) who underwent chemotherapy due to acute lymphoblastic leukemia. Patients were diagnosed with ALL between 1990 and 2010, aged 1–15 years at diagnosis and treated according to the ALL Berlin-Frankfurt-Münster (BFM) 90, 95 and 2002 chemotherapy protocols in ten Hungarian centres.

We stratified our patients in different risk groups according to the following criteria: Low risk (LR) group included children aged 1–6 years who have a white blood cell count of less than 20,000/μl at diagnosis, good prednisone response and no-T-ALL. High risk (HR) group included poor prednisone response, and/or evidence of t(9;22) (or BCR/ABL), and/or evidence of t(4;11) (or MLL/AF4). Medium risk (MR) group included children with no HR or LR criteria. Prednisone responses were determined after 8 and 33 days of induction treatment. The presence of 1,000 blast/μl or more in the peripheral blood on day 8 and/or >5% blast in the bone marrow on day 33 were defined as poor prednisone response.

There is no significant difference in the distribution of age groups or genders or ALL-immunophenotypes between the whole population and our sample-collection.

The 529 control patients (aged 16.1 ± 12.4 years) of the same ethnicity and from the same geographical region as the patients were randomly selected from healthy blood donors and from minor outpatients from the Orthopaedic Department in the Budai Children’s Hospital, and from the Urological Department of Heim Pal Pediatric Hospital, Budapest. None of the controls have had childhood ALL or any other types of cancers previously. In Table 1, some clinical characteristics of ALL patients and controls are presented.

**Table 1:** Some characteristics of ALL patients and controls in the study population

**Characteristics**	**Subgroups**	**ALL**	**Controls**
Total number of subjects		543	529
Gender n (%)	Male	308 (56.7)	305 (57.7)
	Female	235 (43.3)	224 (42.3)
Age at diagnosis, years (mean±SD)		6.4±4.2	16.1±12.4
Number of subjects n (%)	<1 year	8 (1.5)	-
	1-10 years	422 (77.7)	200 (37.8)
	>10 years	107 (19.7)	329 (62.2)
	N/D	6 (1.1)	-
Risk group n (%)	LR	96 (17.7)	-
	MR	309 (56.9)	-
	HR	55 (10.1)	-
Protocols n (%)	90	131 (24.1)	-
	95	250 (46.0)	
	2002	120 (22.1)	-
	N/D	42 (7.7)	
Immunphenotype n (%)	B-ALL	390(71.8)	-
	T-ALL	78 (14.4)	-
Cytogenetics n (%)	Hyperdiploidy	79 (14.5)	-
Overall survival n (%)	516*	414 (85.5)*	-
Event free survival n (%)	516*	418 (81.0)*	-

N/D: no data available; LR: Low risk, MR: Medium risk; HR: High risk, *see details in the text.

All study subjects belonged to the Hungarian (Caucasian) population. Informed consent was requested from the study subjects, or from the parents of patients. The study was conducted according to the principles expressed in the Declaration of Helsinki and was approved by the Hungarian Scientific and Research Ethics Committee of the Medical Research Council (ETT TUKEB; Case No.:8-374/2009-1018EKU 914/PI/08.)

### Candidate gene selection

From the scientific literature we selected 19 candidate genes, which in earlier studies were seemed to be relevant to ALL. We selected genes from the results of GWA studies, candidate gene association studies and from other studies in which the investigated pathways could also be important for ALL. In online databases we searched SNPs for these 19 genes. Altogether 66 SNPs were selected. The selection criterion was: minor allele frequency > 10%. The SNPs were prioritized according to their published role in ALL, and their estimated functionality. In some cases (e.g. in *ARIDB5*, *IKZF1* genes) SNPs were selected, which showed strong linkage disequilibrium with other investigated SNPs in the HapMap database [14]. This could serve as genotyping controls, but during the BN-BMLA evaluation tag SNPs were chosen for the analysis. Table 2 shows information about the selected genes and SNPs.

**Table 2:** Some information about the selected genes and SNPs

**SNP (rs#)**	**Gene**	**Alleles (1/2)**^**a**^	**Position**^**b**^	**Function**^**c**^
rs2066853	*AhR*	G/A	chr7:17345635	missense/Arg>Lys
rs713150*	*AhR*	C/G	chr7:17306682	intron
rs2282885	*AhR*	T/C	chr7:17312139	intron
rs2282883	*AhR*	G/A	chr7:17322872	intron
rs2237297	*AhR*	C/T	chr7:17326119	intron
rs10994982*	*ARID5B*	A/G	chr10:63380110	intron
rs10821936	*ARID5B*	T/C	chr10:63393583	intron
rs7089424	*ARID5B*	T/G	chr10:63422165	intron
rs4948502	*ARID5B*	T/C	chr10:63509423	intron
rs4948496	*ARID5B*	T/C	chr10:63475623	intron
rs4948487	*ARID5B*	A/C	chr10:63339871	intron
rs4506592	*ARID5B*	G/A	chr10:63397193	intron
rs4509706	*ARID5B*	T/C	chr10:63331346	near-gene-5
rs3817074	*BAX*	C/T	chr19:54151024	intron
rs7259013	*BAX*	A/C	chr19:54155096	intron
rs11667351	*BAX*	T/G	chr19:54147966	near-gene-5
rs12457893	*BCL2A*	A/C	chr18:59077141	intron
rs11876772	*BCL2A*	A/G	chr18:58968686	intron
rs2850761	*BCL2A*	A/G	chr18:59117377	intron
rs8092560	*BCL2A*	T/A	chr18:58954673	intron
rs4987845	*BCL2A*	G/A	chr18:58946168	untranslated-3
rs1801018	*BCL2B*	A/G	chr18:59136859	coding-synon/Thr>Thr
rs1893806	*BCL2B*	T/G	chr18:59135944	near-gene-3
rs1799988	*CCR5*	C/T	chr3:46387263	untranslated-5
rs3087253	*CCR5*	T/C	chr3:46393693	near-gene-3
rs11575815	*CCR5*	T/A	chr3:46395174	unknown
rs10403561	*CEBPA*	A/G	chr19:38482342	near-gene-3
rs874966	*CEBPA*	A/G	chr19:38481237	near-gene-3
rs2239633	*CEBPE*	C/T	chr14:22658897	near-gene-5
rs8015478	*CEBPE*	C/A	chr14:22655858	near-gene-3
rs12434881	*CEBPE*	G/A	chr14:22658482	near-gene-5
rs11978267	*IKZF1*	A/G	chr7:50433798	intron
rs4132601	*IKZF1*	T/G	chr7:50438098	untranslated-3
rs6954833	*IKZF1*	A/G	chr7:50425940	intron
rs10235796	*IKZF1*	C/T	chr7:50430131	intron
rs6964969	*IKZF1*	A/G	chr7:50440745	near-gene-3
rs11208538**	*JAK1*	G/C	chr1:65161877	intron
rs310225	*JAK1*	G/A	chr1:65097271	intron
rs12063205	*JAK1*	A/G	chr1:65144640	intron
rs3212713	*JAK3*	G/A	chr19:17816001	intron
rs11888	*JAK3*	T/C	chr19:17796626	near-gene-3
rs2229974	*NOTCH1*	T/C	chr9:138511457	coding-synon/Asp>Asp
rs3124596	*NOTCH1*	A/G	chr9:138521325	intron
rs3124999	*NOTCH1*	C/T	chr9:138515294	intron
rs3124603	*NOTCH1*	T/C	chr9:138529998	intron
rs1800566	*NQO1*	C/T	chr16:68302646	missense/Pro>Ser
rs1469908	*NQO1*	A/G	chr16:68321913	unknown
rs1143684	*NQO2*	T/C	chr6:2955389	missense/Leu>Phe
rs2756075	*NQO2*	C/T	chr6:2949532	intron
rs4149352	*NQO2*	C/T	chr6:2947237	intron
rs2070999	*NQO2*	G/A	chr6:2944728	near-gene-5
rs4149360*	*NQO2*	G/A	chr6:2951806	intron
rs2030171	*STAT1*	G/A	chr2:191577408	intron
rs10208033	*STAT1*	T/C	chr2:191587662	near-gene-5
rs3088307	*STAT1*	G/C	chr2:191537657	untranslated-3
rs12949918	*STAT3*	T/C	chr17:37779799	intron
rs3809758	*STAT3*	G/A	chr17:37725506	intron
rs3816769	*STAT3*	T/C	chr17:37751799	intron
rs17405722	*STAT3*	G/A	chr17:37796027	near-gene-5
rs7217728	*STAT5A*	T/C	chr17:37700927	intron
rs3198502	*STAT5A*	G/T	chr17:37716520	untranslated-3
rs9906933	*STAT5B*	G/A	chr17:37663571	intron
rs4029774	*STAT5B*	A/G	chr17:37682487	near-gene-5
rs703817	*STAT6*	G/A	chr12:55776095	untranslated-3
rs3024979	*STAT6*	T/A	chr12:55774560	intron
rs324015	*STAT6*	G/A	chr12:55776367	untranslated-3

*Failed genotyping, **Monomorphic, ^a^Alleles on the forward strand; 1- major allele; 2-risk allele, ^b^Position according to NCBI Genome Build 36.0, ^c^Function according to CHIP Bioinformatics Tools http://snpper.chip.org/.

### Laboratory methods, genotyping

Genomic DNA from children with leukemia was obtained retrospectively from whole, peripheral blood taken in remission phase using by QIAmp DNA Blood Maxi Kit (Qiagen, Hilden, Germany) according to the manufacturer’s instructions. Before DNA extraction the normal leukocyte cell count was checked by FACS. We used only those samples where the normal cell count was over 5 G/l. DNA from healthy control samples was isolated from whole peripheral blood using iPrep PureLink gDNA Blood Kit, iPrep Purification Instrument (Invitrogen, Life Technologies Co., Grand Island, USA). Genotyping was carried out by Sequenom iPLEX Gold MassARRAY technology at the McGill University and Génome Québec Innovation Centre, Montréal, Canada. Only those SNPs were included in the analysis, which have a genotyping call rate over 90%.

### Frequentist-based statistical analysis

Allele frequencies were calculated by allele counting. Hardy-Weinberg equilibrium was tested by using a χ^2^ goodness-of-fit test with an acceptable cut off value of p ≥ 0.01 (online application: [15]).

The distributions of alleles and genotypes in cases and controls were compared by means of the χ^2^ test [15]. Association between the candidate SNPs and the susceptibility to childhood acute lymphoblastic leukemia was tested using gender adjusted logistic regression in the case–control data performed by IBM SPSS Statistics software (version 19.0). Confidence interval was calculated at the 95% level (C.I. 95%). Multiple testing corrections were performed using the Benjamini-Hochberg false discovery rate (FDR) method with type I error rate of 1%.

Haplotype analyses were carried out by Haploview version 4.2 software (Broad Institute, Cambridge, Maine, USA). Odds ratios for haplotypes were calculated by MedCalc 12.1.1 software (MedCalc Software, Mariakerke, Belgium). Survival analyses were performed using Kaplan-Meier method. The log-rank test was applied for evaluating the association between survival and categorized parameters, as risk group, gender, and study-protocols. The statistical tests were carried out by IBM SPSS Statistics software, version 19.0.

### Bayesian Network based Bayesian Multilevel Analysis

A Bayesian Network (BN) is a probabilistic graphical model that represents the conditional dependencies of a set of random variables with a directed acyclic graph (DAG). A BN can efficiently describe the joint probability distribution of the variables. A node of the graph represents a variable and an edge represents a direct dependency between the corresponding variables.

Structure learning is finding a DAG that best describes the dataset. However, in most cases, where the amount of available data is modest relative to the number of variables, usually there are many DAGs that have non-negligible a posteriori probabilities. Even in these cases, there might be certain structural features, e.g. the presence of an edge, which we can extract reliably. The Bayesian learning framework enables us to estimate a posteriori probability of a certain feature *f* as follows

(1) $$P\left(f|D\right)=\sum_{G}f\left(G\right)P\left(G|D\right)$$

where *G* represents a BN structure, *D* is the dataset, and *f*(*G*) is an indicator function of the presence of *f* in *G*, i.e. it is 1 if the feature holds in *G* and 0 otherwise.

We use Bayes’ theorem to calculate *P*(*G*|*D*), and we have that $P\left(G|D\right)\propto P\left(D|G\right)P\left(G\right)$. Assuming that there are no missing values in the dataset *D*, the variables are multinomial with a Dirichlet parameter prior for every possible instantiations of their parents, and the prior *P*(*G*) satisfies some general constraints, then the term *P*(*D*|*G*) (i.e. the marginal likelihood of the data) can be computed efficiently in a closed form [16]. The other term, *P*(*G*) is the a priori probability of a structure *G*. We use uniform prior over structures in our experiments and the K2 hyperparameter prior, as proposed by Cooper and Herskovits [16].

Because the number of BN structures is super-exponential in the number of nodes, exact summation of all possible structures *G* is computationally intractable. We use Metropolis-coupled Markov Chain Monte Carlo (MC^3^) methods [17] over the space of DAGs for the approximation of Eq.1. We run the Metropolis-coupled Markov Chain sampler with a burn-in period of 2x10^7^ steps and then collect 6x10^7^ samples (i.e. network structures). We restrict the space of the possible structures limiting the number of parents per node to 4. To ensure the convergence of the posterior of the structural features estimated by the MCMC, we use Gelman-Rubin R-scores [18] and Geweke Z-scores [19]. All reported posteriors had an R-score below 1.05, and Geweke Z-scores also confirmed convergence.

**Figure 1:**
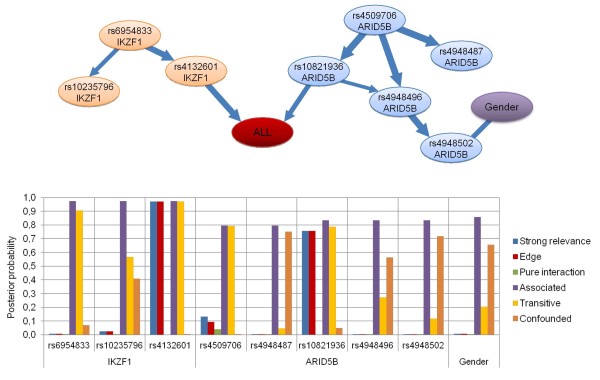
**Illustration of different dependency relations between certain SNPs in *ARID5B *and *IKZF1 *genes, gender and ALL susceptibility.** Top panel: The “averaged structure” of the Bayesian networks including ALL susceptibility (red node), gender (purple node) and the SNPs of *ARID5B* (blue nodes) and *IKZF1* (orange nodes). The width of the edges is proportional to their a posteriori probability. The probability of the edges is computed by averaging over the Bayesian networks visited by the MCMC process. See Methods. Bottom panel: The posterior probability of strong relevance (blue columns), edge (direct strong relevance, red columns), pure interaction (green columns), association (purple columns), transitive association (yellow columns) and confounded association (brown columns) of the variables to ALL susceptibility according to the BN-BMLA method.

We computed a posteriori probabilities for structural features summarized in Table 3, and as an illustration see Figure 1.

**Table 3:** Structural features that indicate different dependence types between the variables

**Relation**	**Abbreviation**	**Graphical**
**Pairwise features**		
Direct causal relevance	DCR(X,Y)	There is an edge between X and Y
Transitive causal relevance	TCR(X,Y)	There is directed path between X and Y
Confounded relevance	ConfR(X,Y)	X and Y have common ancestor
Association	A(X,Y)	DCR or TCR or ConfR
Pure interactionist relevance	PIR(X,Y)	X and Y have common child
Strong relevance	SR(X,Y)	PIR or DCR
**Relevance of variable sets**		
Strong relevance	MBS(Y)	The set consisting of Y’s parents, its children, and the other parents of its children (the Markov Blanket Set of Y)
**Interaction models of relevant variables**		
Strong relevance	MBG(Y)	The subgraph that includes the nodes in the MBS and the incoming edges into Y and into its children (the Markov Blanket Subgraph of Y)

The current implementation of our method deals with only discrete variables. Continuous covariates have to be discretized first in order to include them in the BN-BMLA analysis. In this paper, we use only discrete variables (SNPs, gender, case/control status, 5-year survival).

### Computing redundancies and interactions

In a post-processing step, we can compute the a posteriori probability of the strong relevance of any set of variables with a given size *k*[9,10]. The a posteriori probability of the sub-relevance of a *k*-sized set *s* is denoted with a special notation as follows $\underset{Â¯}{p}\left(s|D\right)=P\left(MBS\left(Y\right)=s|D\right)+\sum_{s\subset s^{\prime }}P\left(MBS\left(Y\right)=s^{\prime }|D\right)$, where the terms are the MBS posterior of the set *s* and the MBS posteriors for all its superset.

We calculate the interaction or redundancy [10] of a variable set $\left\{X_{i_{1}},\ldots ,X_{i_{n}}\right\}$ by computing $R=\frac{\underset{Â¯}{p}\left(\left\{X_{i_{1}},\ldots ,X_{i_{n}}\right\}|D\right)}{\prod_{k=1}^{n}\underset{Â¯}{p}\left(\left\{X_{i_{k}}\right\}|D\right)}$.

Where the numerator is the a posteriori probability that the variable set is strongly relevant with respect to the target variable, and the denominator is the product of the a posteriori probability of the strong relevance of the variables in the set.

If R equals 1, this means that the approximation of the strong relevance of the set under an independence assumption (i.e. the denominator) is equal to the real a posteriori probability of the set (i.e. the numerator). In this case, the variables in the set are independently relevant (i.e. they are independently present or absent in the Markov Blanket set of the target variable).

If *R* differs from 1, it indicates an interaction (*R* > 1) or a redundancy (*R* < 1). The corresponding Interaction Ratio is then =ln(*R*), and the Redundancy Ratio is *RR* = –ln(*R*).

## Results

### SNPs associated with ALL risk

As the blood sample was taken retrospectively and in remission, and only those samples were used where the normal leukocyte cell count was over 5 G/l, practically no tumour cells were in our samples, thus we investigated only normal cells and germline polymorphisms.

From the 66 genotyped SNPs one SNP was monomorphic (minor allele frequency = 0), and three had poor genotyping results (poor genotype clusters or low call rates), leaving 62 SNPs for frequentist and BN-BMLA analyses.

Minor allele and genotype frequencies in controls and ALL patients are presented in Additional file 1. The results of the statistical analysis of all genotyping data are presented in Additional file 2.

The differences in allele and genotype frequencies between ALL cases and controls were nominally significant (P < 0.05) for 20 SNPs. But, when gender adjusted logistic regression analysis with false discovery rate of (FDR(α)=) 1% significance threshold was calculated, the differences remained significant (P < 3.42E-04) only in cases of 6 SNPs in two genes (rs10821936, rs7089424 and rs4506592 in *ARID5B*, and rs6964969, rs11978267 and rs4132601 in *IKZF1,* as shown in Table 4). These results indicate that these SNPs are associated with increased susceptibility to ALL with odds ratios between 1.4 and 1.5. Then we analyzed whether the number of risk alleles of each SNP influenced the susceptibility to ALL, and found that in all cases the homozygous states are associated with higher risk (OR between 1.9 - 2.1) than the carrier status, or heterozygous states.

**Table 4:** Summary of the significant results of logistic regression analyses

**Gene**	**SNP**	**ALL**			**B-ALL**			**T-ALL**			HD-ALL*		
		**P-value**	**OR**	**95% C.I.**	**P-value**	**OR**	**95% C.I.**	**P-value**	**OR**	**95% C.I.**	**P-value**	**OR**	**95% C.I.**
*IKZF1*	rs6964969	**1.67E-05**	1.50	1.25-1.80	**1.17E-07**	1.70	1.40-2.08	1.88E-01	0.76	0.50-1.14	8.72E-02	1.36	0.96-1.94
*IKZF1*	rs11978267	**2.46E-05**	1.50	1.24-1.79	**2.97E-07**	1.68	1.38-2.05	2.11E-01	0.77	0.51-1.16	5.08E-02	1.42	1.00-2.02
*IKZF1*	**rs4132601**	**1.69E-05**	1.50	1.25-1.80	**2.22E-07**	1.69	1.38-2.06	3.46E-01	0.83	0.55-1.23	9.13E-02	1.36	0.95-1.94
*ARID5B*	**rs10821936**	**7.31E-05**	1.43	1.20-1.71	**1.95E-05**	1.53	1.26-1.85	3.90E-01	1.17	0.82-1.66	5.72E-03	1.61	1.15-2.27
*ARID5B*	rs7089424	**1.17E-04**	1.42	1.19-1.69	**2.68E-05**	1.52	1.25-1.84	4.14E-01	1.16	0.81-1.65	6.70E-03	1.60	1.14-2.24
*ARID5B*	rs4506592	**1.72E-04**	1.40	1.18-1.67	**3.35E-05**	1.51	1.24-1.83	6.70E-01	1.08	0.76-1.54	1.24E-02	1.55	1.10-2.19
*STAT3*	rs3816769	3.76E-02	0.83	0.69-0.98	4.23E-02	0.82	0.67-0.99	5.82E-01	0.91	0.64-1.29	**1.34E-04**	0.62	0.49-0.79
*STAT3*	**rs12949918**	5.15E-02	0.84	0.71-1.00	6.37E-02	0.84	0.69-1.01	5.54E-01	0.90	0.64-1.27	**2.32E-04**	0.64	0.50-0.81

*Hyperdiploid ALL; Tag SNPs are bold, as well as P-values, which reached the p < 3.42E-04; FDR(α)= 1% significance threshold.

We calculated the linkage disequilibrium (LD) coefficients between the different SNPs (Additional file 3), and found that in both genes, the significantly associated SNPs were in strong linkage with each other. This means that there is only one, but strong signal in each gene. We also evaluated the effect of different haplotypes on the risk to ALL. We found two haplotypes (Additional file 4), which influenced the susceptibility to ALL in the *IKZF1* gene. But the odds ratio associated with the haplotypes, were not higher than in the case of individual SNPs. Interestingly, however, a haplotype containing the major alleles of the two individually associated SNPs (rs4132601, rs6964969) in the *IKZF1* gene shows a slight protection against ALL (OR = 0.73 (0.62-0.86); P = 3.0E-04).

Subsequently, it was investigated whether these SNPs influence the clinical characteristics of ALL. As can be seen in Table 4 all the SNPs which were associated with ALL, increased the risk of B-cell ALL, but not of T-cell ALL. When the hyperdiploid ALL (≥ 50 chromosomes) was considered, two SNPs in the *STAT3* gene (rs3816769, rs12949918) showed decreased risk to this clinical subtype of the disease. As these SNPs are in LD with each other (Additional file 3), it also corresponds to one signal. Interestingly, these SNPs do not influence the risk to ALL in general.

We also investigated whether the gender of the patients influence the effect of the SNPs, but found no such effect.

### Survival of ALL patients

We also investigated which factors influence the 5- year-survival of the patients. We have data about the survival rate in 516 cases (95% of all patients). Our sample set contains similar rate of relapsed patients to what was observed in the whole population [8]. However, the rate of died patients is lower in our study population (p < 0.001). Patients who died during the chemotherapy due to therapy resistant progressive disease or due to infections or toxicities of therapy are underrepresented in our sample.

**Figure 2:**
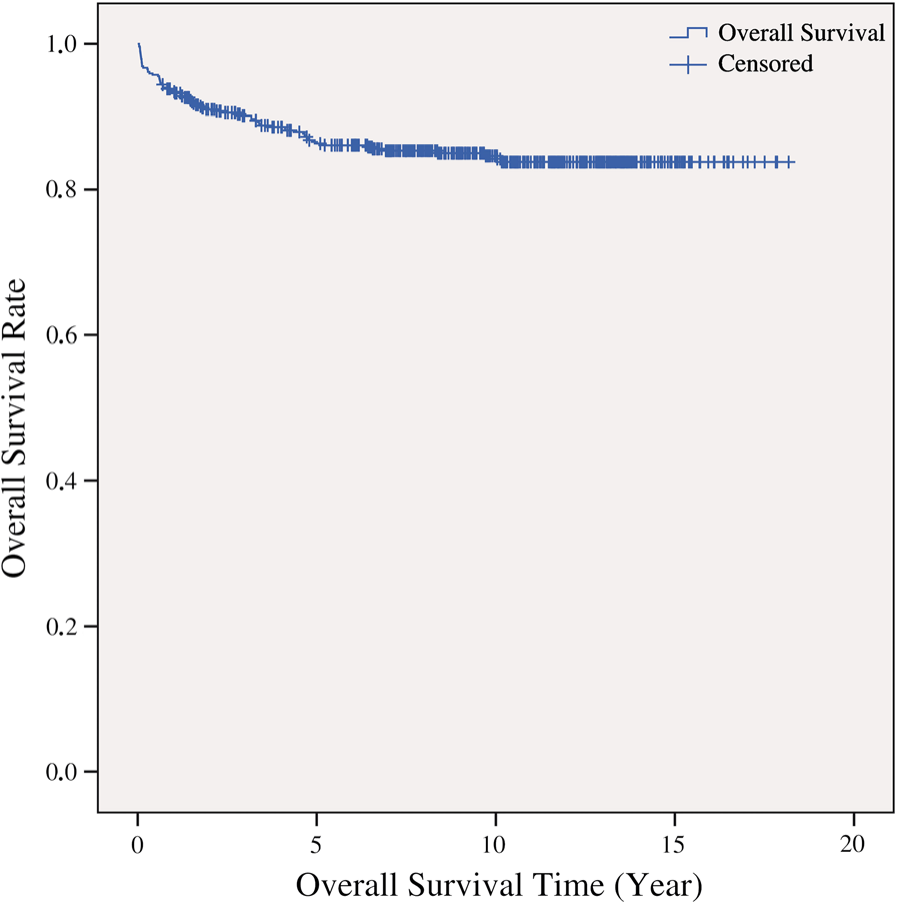
Overall survival rate of the ALL patients by survival time.

The overall survival rate was 85.5% (n = 441) in our childhood ALL population (Figure 2).

**Figure 3:**
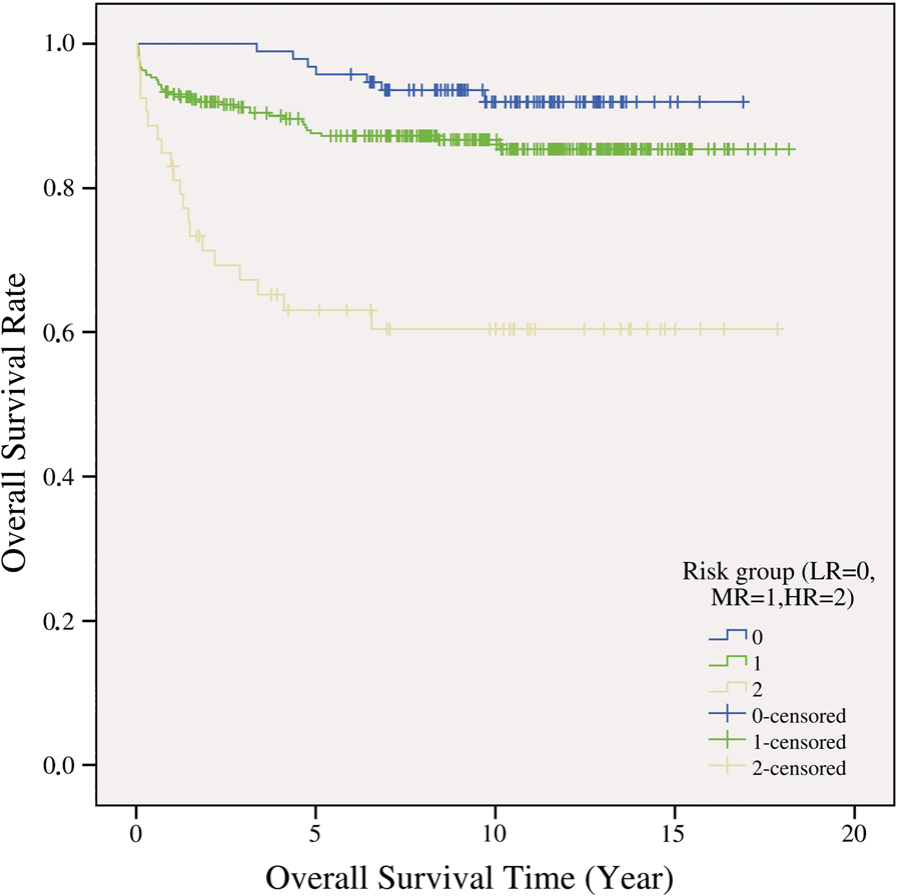
Overall survival rates according to the risk groups (LR = low risk; MR = medium risk; HR = high risk).

There was no significant difference between sex groups and study protocols in overall survivals. There was significant difference between risk groups in overall survivals (p = 1E-7). The survival rates were 92.6% in the low, 87.0% in the medium and 62.3% in the high risk groups (Figure 3).

The event-free survival rate was 81.0%. There was no significant difference between sex groups and study protocols in event-free survivals. There was significant difference between risk groups in event-free survivals (p = 1E-7). The survival rates were 90.4% in the low, 82.6% in the medium and 60.4% in the high risk groups.

### SNPs influencing the survival of the ALL patients

We investigated, whether the SNPs in our study influenced the survival of the patients. Altogether 4 SNPs in 3 genes (rs10403561 and rs874966 in *CEBPA*, rs3024979 in *STAT6*, rs11667351 in *BAX*) were nominally significant in this respect, but none of them reached our significance threshold. Among these results the rs11667351 in the *BAX* gene showed the strongest association and gave the lowest p value (P = 0.001; Additional file 5). The detailed results of these SNPs can be seen in Additional file 6. The overall and the event-free survival did not differ in this respect.

### BN-BMLA method for ALL susceptibility

Besides evaluating our results with the traditional frequentist-based methods, we also analyzed them with our newly developed BN-BMLA method. Because the results and their interpretation differ from those of the standard statistical methods, here, in the results section we also give some explanations for their understandings.

As the results of the BN-BMLA are strongly influenced if the studied SNPs are in LD with each other, we selected 48 tag SNPs. Gender was also involved in the analysis as a variable. For each variable (SNPs and gender) we calculated posterior probability for strong relevance. Posterior values are between 0 and 1, where 0 indicates no relevance, 1 indicates 100% relevance between a predictor and a target variable. According to our previous considerations [12,13], a variable is relevant when its posterior of strong relevance is greater than or equal to 0.5. Above 0.75 the relevancy is regarded as convincing, between 0.5 and 0.75 as moderate.

The results of the analysis of ALL risk are presented in Additional file 7. The most relevant SNPs and genes (i.e. with high posteriors for strong relevance) according to the BN-BMLA are presented in Table 5. In case of ALL susceptibility, the most relevant SNPs are rs10821936 in *ARID5B* and rs4132601 in *IKZF1*. The probability that these SNPs are directly associated to ALL is 0.76 for rs10821936 and 0.97 for rs4132601, respectively. Both of these direct associations are even more probable in case of the B-cell lineage sample group (0.95 and 1.0 for rs10821936 and rs4132601, respectively). However, in case of the T-cell lineage sample group, the probability of the direct relevance of these SNPs is very low, namely, around 0.02. As the B-cell lineage is lot more frequent, the high probability of the strong relevance of these two SNPs in case of ALL susceptibility is probably due to their strong relevance in B-cell lineage.

**Table 5:** Posterior probabilities of strong relevance of some variables in different groups

**Phenotype**	**ALL**	**B-ALL**	**T-ALL**	**HD-ALL**	**Risk group**
Gender	0.01	0.06	0.85*	0.07	0.08
rs10821936 (*ARID5B*)	0.76*	0.95*	0.02	0.14	0.05
rs12949918 (*STAT3*)	0.01	0.00	0.01	0.60*	0.00
rs17405722 (*STAT3*)	0.23	0.24	0.21	0.37	0.09
rs12457893 (*BCL2*)	0.02	0.01	0.24	0.57*	0.05
rs1893806 (*BCL2*)	0.02	0.00	0.02	0.01	0.42
rs3212713 (*JAK3*)	0.03	0.11	0.17	0.56*	0.01
rs3087253(*CCR5*)	0.03	0.10	0.15	0.56*	0.00
rs2282883 (*AhR*)	0.01	0.00	0.11	0.08	0.42
rs2066853 (*AhR*)	0.06	0.04	0.25	0.34	0.04
rs4132601 (*IKZF1*)	0.97*	1.00*	0.02	0.02	0.00

*relevant posteriors (Posterior probability > 0.5); HD-Hyperdiploid.

In the T-cell lineage, only the gender of the patient has a high probability of direct association to ALL susceptibility (0.85), where males have significantly higher odds of developing ALL than women (OR = 2.28, C.I. 95%: 1.32 – 3.93).

In the hyperdiploid sample group, the probabilities of the most relevant SNPs are moderate. Namely, rs12949918 in *STAT3* has 0.6, rs12457893 in *BCL2* has 0.57, rs3212713 in *JAK1* has 0.56, and rs3087253 in *CCR5* has 0.56 probability of being strongly relevant to the hyperdiploid phenotype.

### Detailed characterization of association relations

In the context of genetic association studies, strongly relevant variables with respect to a phenotype represent the genetic and phenotypic factors that directly influence the phenotype (e.g. disease susceptibility). However, the standard concept of pairwise association is not identical to the concept of strong relevance.

The association of a genetic variant to a phenotypic feature can have multiple types (Figure 1). First, the causal SNP has a direct influence on the phenotype. In this case the probability of the existence of a direct edge between the causal SNP and the phenotypic variable will be high. Second, due to linkage disequilibrium or evolutionary patterns, the genetic factors are often dependent on each other. Because of this dependency, univariate association tests can not necessarily make the distinction between the true causal SNP and the SNPs that are strongly linked to it. In these cases, the association is mediated by the causal SNP, and there exists a path between the associated SNP and the phenotypic variable with high probability. The association is transitive if the path is directed, and confounded if it is not. Third, if a SNP has no main effect on the phenotype, but has an epistatic effect along with another factor, then this SNP is not associated (according to the standard concept of association), but strongly relevant to the phenotype (i.e. it is in pure interaction with the phenotype).

We computed the a posteriori probability of the different association types with respect to ALL susceptibility in all sample groups (Additional file 7). Here, we present some examples how the results in Additional file 7 can be interpreted. The probability of the association of the SNPs in *ARID5B* (rs4509706, rs4948487, rs10821936, rs4948496, rs4948502) to ALL susceptibility are equal or greater than 0.8, but only rs10821936 can be stated as directly relevant, because its posterior of strong relevance is 0.76 and the posterior of strong relevance is below 0.13 in case of all other SNPs, which indicates their non-causal, non-functional role. The situation is same in case of the SNPs in the *IKZF1* gene. The association of the SNPs rs6954833, rs10235796, and rs4132601 in *IKZF1* to ALL susceptibility is highly probable (0.97), but only rs4132601 has a high probability (0.97) of being strongly relevant. These effects are more expressed in the B-cell lineage sample group.

The probability of pure interaction is very low (below 0.1) in case of all SNPs in ALL susceptibility and B-cell lineage sample groups. However, in other sample groups, there are some SNPs with low probability of being in pure interaction to the phenotype, e.g. 0.43 in case of rs3212713 in *JAK3* in the hyperdiploid sample group, and 0.42 in case of rs2282883 in *AHR* when the target variable was the risk group of the patients.

### Redundancies and interactions

**Figure 4:**
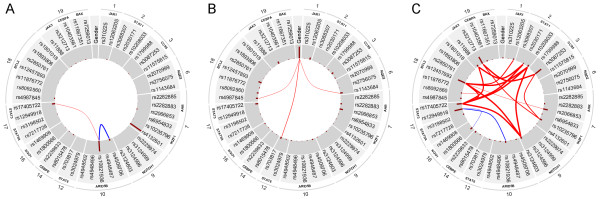
**Redundancies and interactions according to the BN-BMLA method.** The figure shows the magnitude of redundancies (blue curved lines) and interactions (red curved lines) between the variables in the whole dataset (i.e. ALL susceptibility, **A** panel), in the T-cell lineage sample group (**B** panel) and in the hyperdiploid sample group (**C** panel) according to the BN-BMLA method. See Methods for the computation of interaction and redundancy. The width of the curved lines is proportional to the strength of the effect. The a posteriori probability of the strong relevance of the variables is proportional to the length of the dark red columns next to the variable in the inner gray colored ring. The corresponding genes and chromosomes of the SNPs are shown on the outer ring.

The Bayesian analysis offers a principled way to compute the measure of interaction or redundancy of two (or more) SNPs. In this context, interaction means that two SNPs tend to occur together more often in the hypotheses of the strongly relevant variable sets than it is expected (i.e. the joint strong relevance of the two SNPs is more probable than it can be approximated from their univariate relevance). On the other hand, redundancy means that two SNPs are somewhat interchangeable in the hypotheses of the strongly relevant variable sets (i.e. the probability of the joint strong relevance of two SNPs is lower than it is expected). We computed the redundancies and interactions between all variables in case of all sample groups. In Figure 4, we present the results in a graphical form in case of the whole dataset (ALL susceptibility), the T-cell lineage patient group and the hyperdiploid patient group. The analysis did not result in any interactions or redundancies in case of the other sample groups.

In case of ALL susceptibility, rs10821936 in *ARID5B* and rs17405722 in *STAT3* showed a weak interaction (Interaction ratio (IR) = 0.15) and two SNPs in *ARID5B*, namely rs10821936 and rs4509706 showed a moderate redundancy (Redundancy ratio (RR) = 0.33). *ARID5B* and *IKZF1* (the two genes that have the highest posterior of strong relevance to ALL susceptibility) showed no interaction or redundancy with each other.

In case of T-cell lineage sample group, the gender showed a weak interaction with three SNPs in three genes, namely rs703817 in *STAT6* (IR = 0.16), rs4987845 in *BCL2* (IR = 0.1), and rs1143684 in *NQO2* (IR = 0.11). This latter could be confirmed by logistic regression analysis, as well. This indicated, that male status increased the risk of T cell ALL, but carrying an allele of rs1143684 slightly decreased the risk (interaction tag: p = 0.039, OR = 0.565, C.I. 95%: 0.33 – 0.97).

In case of the hyperdiploid sample group, several overlapping components (i.e. sets of SNPs) were found that showed strong interaction. The component with the highest interaction ratio (IR = 3.06) consists of four SNPs in four genes, namely rs17405722 in *STAT3*, rs12457893 in *BCL2*, rs10208033 in *STAT1*, and rs3124603 in *NOTCH1*. The component with the second highest interaction ratio (IR = 2.72) consists of three SNPs in three genes, namely rs17405722 in *STAT3*, rs11888 in *JAK3*, and rs2030171 in *STAT1*. The third component (IR = 0.86) consists of three SNPs, rs3212713 in *JAK3*, rs3087253 in *CCR5*, and rs10235796 in *IKZF1*. Since these datasets are relatively small, exact characterization of these effects needs further validation.

### Survival analysis with BN BMLA

**Figure 5:**
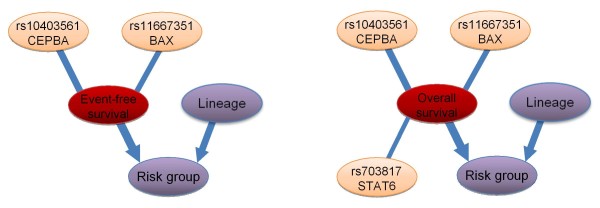
**Subgraphs of the strongly relevant variables in event-free and overall survival.** Left: The “averaged structure” of the subgraphs of the strongly relevant variables in event-free survival according to the BN-BMLA method. Right: The “averaged structure” of the subgraphs of the strongly relevant variables in overall survival according to the BN-BMLA method. The width of the edges is proportional to their a posteriori probability. Edges are shown only if their a posteriori probability exceed 0.5. The probability of the edges is computed by averaging over the Bayesian networks visited by the MCMC process. See Methods. Target variables are indicated with red color, phenotypic variables with purple color, and SNPs with orange color.

All calculated posterior probabilities in event-free survival (EFS) and overall survival (OS) are presented in Additional file 8, and the most relevant variables in Table 6. In both cases, lineage (B- or T-cell) and risk group were found to be strongly relevant to the target variable (event-free and overall survival indicator) with high probability. However, it is more probable that lineage is in pure interaction (P = 0.68 in EFS) than it has a direct relevance (P = 0.09 in EFS). Its effect is mediated by the risk group, thus lineage is important only if risk group is known (Figure 5).

**Table 6:** Posterior probabilities of the most relevant variables in event-free and overall survival

	**Event-free survival**			**Overall survival**		
	**Strong relevance**	**Edge**	**Pure interaction**	**Strong relevance**	**Edge**	**Pure interaction**
Lineage (T- or B-cell)	0.77	0.09	0.68	0.79	0.12	0.67
Risk group	0.99	0.99	0.00	1.00	1.00	0.00
rs4509706 *(ARID5B)*	0.11	0.03	0.08	0.54	0.26	0.28
rs703817 *(STAT6)*	0.34	0.32	0.02	0.67	0.65	0.02
rs10403561 *(CEBPA)*	0.63	0.63	0.00	0.62	0.62	0.00
rs11667351 *(BAX)*	0.79	0.78	0.01	0.87	0.87	0.01

In both cases, the strongly relevant genetic factors with the highest probability are rs11667351 in *BAX* (0.79 in EFS, and 0.87 in OS), and rs10403561 in *CEBPA* (0.63 in EFS, and 0.62 in OS). Besides, a SNP in *STAT6*, namely rs703817 can be indicated as strongly relevant in case of OS (P = 0.67), but its probability is lower in case of EFS (P = 0.34).

**Figure 6:**
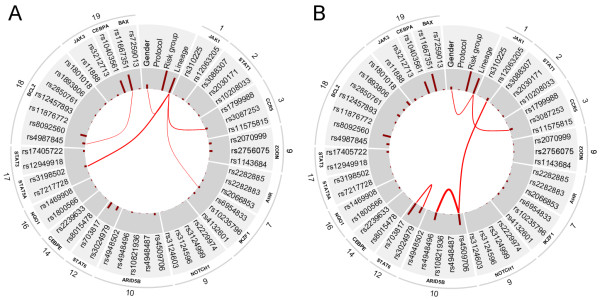
**Interactions in event-free and overall survival according to the BN-BMLA method.** The figure shows the magnitude of interactions (red curved lines) between the variables in the event-free survival (**A** panel), and in the overall survival (**B** panel) according to the BN-BMLA method. See Methods for the computation of interaction. The width of the curved lines is proportional to the strength of the effect. The a posteriori probability of the strong relevance of the variables is proportional to the length of the dark red columns next to the variable in the inner gray colored ring. The corresponding genes and chromosomes of the SNPs are shown on the outer ring.

We computed the redundancies and interactions between all variables in both survival types, as well. The results are shown in Figure 6.

## Discussion

In this study we presented our results of a candidate gene association study in childhood acute lymphoblastic leukemia evaluated by traditional frequentist-based methods and our newly developed BN-BMLA method.

According to the frequentist-based evaluation, among the successfully genotyped 62 SNPs in 19 genes, 6 SNPs in 2 genes associated with increased susceptibility to ALL. But, as the SNPs in both genes were in LD, this corresponded to one signal in each gene, namely in *ARID5B* and *IKZF1*. Both associations were specific for B-cell ALL, and in all loci the effect on risk was dose-dependent, as homozygous states associated with higher risks.

ARID5B belongs to a family of transcription factors important in embryonic development, cell type–specific gene expression, and cell growth regulation. SNPs in the gene have been found associated with ALL in several previous genome wide and candidate gene association studies and in different populations [3,4,6,20]. In some studies the associations were restricted to B-hyperdiploid ALL and to males. In our study no such difference was found between boys and girls, and the SNPs were not associated with hyperdiploidy, but the important role of the gene in B-cell ALL susceptibility was confirmed. It must be added, however, that all of the associated SNPs in the *ARID5B* gene are in intron, and presently it is not known, how they influence the risk to ALL.

SNPs in the *IKZF1* gene were identified by independent genome wide association studies in Caucasian children; although later in some studies and in some populations the association was not confirmed [3,4,21]. *IKZF1* (encoding the lymphoid transcription factor IKAROS) is deleted in approximately 80% of the Philadelphia chromosome–positive ALL with constitutively active BCR-ABL1 tyrosine kinase [22,23]. Furthermore, Ikaros proteins are master regulators of lymphocyte development, thus *IKZF1* is a good candidate gene for ALL. Earlier, one of the SNPs, the rs4132601, which showed an association with increased susceptibility to ALL, and influenced in an in vitro system the expression level of the gene in a dose-dependent fashion, with lower expression being associated with the risk alleles [4]. Since human and mouse studies suggest that diminished expression of *IKZF1* interrupts lymphocyte development, creating conditions that maintain the rapidly dividing lymphoblasts that characterize ALL, the lower expression associated with the rs4132601 might contribute to the increased risk to the disease.

SNPs in the *STAT3* gene were found to decrease the risk to hyperdiploid ALL. The signal transducer and activator of transcription protein 3, encoded by *STAT3*, has been identified as a regulator of cell survival after exposure to apoptotic signals [24-26]. STAT3 serves, among others, as a substrate for SYK tyrosine kinase. SYK is capable of associating with and phosphorylating STAT3 in human B-lineage leukemia/lymphoma cells challenged with oxidative stress [27]. Inhibition of SYK with a small molecule drug candidate prevented oxidative stress-induced activation of STAT3 and overcame the resistance of human B-lineage leukemia/lymphoma cells to apoptosis. The decreased risk associated with the SNPs in the *STAT3* gene in our study correlates with the results of other studies, e.g. the rs12949918 SNP was found to be associated with decreased susceptibility to different malignancies, like risk to B-cell non-Hodgkin lymphoma or renal cell carcinoma [28]. According to *in vitro* studies, the SNP affects *STAT3* mRNA levels, with the minor allele having a lower STAT3 expression [29]. Presently it is not known how it might influence the risk to hyperdiploid ALL.

We also investigated whether the SNPs influence the survival of the disease. It must be noted, however, that the rate of died patients is lower in our study population (p < 0.001), than in the whole Hungarian ALL population (see Methods), thus our results could be biased in this respect. In the frequentist-based evaluation, the rs11667351 in the *BAX* gene showed the strongest association and gave the lowest p value (P = 1E-3), but it still did not reach the significance threshold (P ≤ 3.42E-4).

Next, we evaluated our results with the BN-BMLA method. Earlier, we applied this method to evaluating a partial genome screening in asthma [13]. In that study the frequentist-based method identified two genes for asthma susceptibility (*FRMD6* and *PTGDR*), while the BN-BMLA identified 3 additional genes. When we analyzed the cause of this difference, it turned out that the other 3 genes indirectly associated with asthma risk, i.e. in different gene-gene interactions. Then, as BN-BMLA is also capable of analyzing multiple targets, we involved additional phenotypic, target variables in the analysis. In this case the BN-BMLA identified 3 additional genes. The SNPs in these genes influenced the susceptibility to asthma through other target variables, like rhinitis, IgE, or eosinophil levels. As all of these phenotypic variables are in strong association with asthma, association with these might also cause association with the disease. This latter is called transitive associations.

In the present study we could not involve additional target variables in the evaluation, as there are no common known phenotypic characteristics in controls, which significantly change the risk to ALL. The BN-BMLA could confirm the association of SNPs in the *ARID5B* and *IKZF1* to B-cell ALL with high posterior probability. Additionally, however, as explained in the detailed characterization of association relation, the results of the analysis gave additional information about the nature of the relations between the SNPs and the disease. In this case no strongly relevant interactions were found, but the analysis suggested several weak interactions. E.g. in case of ALL susceptibility, rs10821936 in *ARID5B* and rs17405722 in *STAT3* showed a weak interaction, and in case of T-cell lineage sample group, the gender showed a weak interaction with three SNPs in three genes (Figure 4). Interestingly e.g., as it is also known from the scientific literature, the male gender increased the risk of T-cell ALL, but carrying an allele of rs1143684 in the *NQO2* gene slightly decreased the risk. The BN-BMLA is also capable of calculating redundancy of the variables. E.g. in the present study the rs10821936 and rs4509706 SNPs in *ARID5B* gene, showed a moderate redundancy, i.e. their effects are interchangeable with each other. It is also an important finding, that there are no interactions or redundancies between the two most relevant genes, *ARID5B* and *IKZF1*. This is also a confirmation of the results of other studies where no interactions were found between the two genes [30].

Several interactions have been detected in hyperdiploid ALL (Figure 4 C). Although the number of patients in this group is relative low, thus the results must be handled with some reservations, most of these interactions are biologically plausible. E.g. in our analysis a strong interaction was found among SNPs in the *NOTCH1*, *STAT1*, *STAT3* and *BCL2* genes. In the scientific literature it is known that *STAT3* is activated in the presence of active Notch. Notch-IC stable transfectants increased *STAT1*-dependent transcription in response to IFN-gamma. In a zebra fish model of human *NOTCH1*-induced T-cell leukemia, the leukemia onset was dramatically accelerated when the transgenic fish was crossed with another line over expressing the zebra fish *Bcl2* gene, indicating synergy between the Notch pathway and the *Bcl2*-mediated antiapoptotic pathway [31-33]. All of these results suggest that the pathways represented by these genes are overlapping and there are interactions between them.

Analyzing the effects of the variables on the survival of the patients with BN-BMLA resulted in two SNPs in two genes with strong relevance. The relevance of the rs11667351 SNP in the *BAX* gene was convincing (posterior probability > 0.75), while that of the rs1040356 in the *CEBPA* gene was moderate. As can be seen in Figure 6, the SNP in the *BAX* gene was also involved in an interaction with a SNP in the *BCL2* gene. This interaction is biologically plausible, as there are a lot of known interactions between the products of the two genes [34]. E.g. BCL2 prevents BAX/BAK oligomerization, and BCL2 binds to and inactivates BAX. There are also data, although inconclusive, about the role of BAX in the relapse of children with ALL. High levels of BAX protein have been associated with an increased probability of relapse in one study, while in another study both BAX expression levels and the BAX/BCL2 ratio were significantly lower in samples at relapse compared to samples at initial diagnosis [35,36]. In our study, children homozygous to the minor allele of the rs11667351 SNP had a very poor survival rate (40%, Additional file 5 and Additional file 6). In a study, this variant was associated with lower *BAX* mRNA in lymphocytes. It must be added, however, that the number of patients homozygous to the minor allele of the rs11667351 was very low in our population.

The protein encoded by *CEBPA* is a transcription factor which can bind as a homodimer to certain promoters and enhancers. Its mutations have been found to be implicated in acute myelogenous leukemia with favorable prognosis [37]. In our study minor allele carrier status showed better survival rate, than major allele homozygotes, suggesting certain concordance between the two observations.

The BN-BMLA also detected known connections between risk groups, lineage and survival, but also revealed some possible interactions between certain variables like gene-gene, lineage-gene or lineage-gender (Figure 6).

Evaluating the effect of SNPs on the survival rate of the patients resulted in some discrepancies between the two methods. The frequentist-based method detected only nominally significant associations, which, according to the accepted rules, have to be rejected, while the BN-BMLA, especially in the case of the *BAX* gene found strong, convincing relevancy. It is generally accepted, that the frequentist-based methods cannot properly handle the multiple testing problem. To avoid type I error, sometimes the frequentist methods are too conservative and unable to detect weak effects or interactions. The findings of the BN-BMLA are biologically plausible, but additional studies are needed to confirm these results.

In the present paper we show the ability of BN-BMLA to evaluate a candidate gene association study. As can be seen from the results, the advantage is not that the BN-BMLA can detect more relevant variables, but the Bayesian networks offer a rich language for the detailed representation of types of relevance, including direct and indirect aspects. Additionally, Bayesian statistics offer an automated and normative solution for the multiple hypothesis testing problems [11]. In this study we could not utilize the full potential of the BN-BMLA, since we could not include multiple targets present also in controls, we did not have data from different sources (e.g. from gene expression analysis) and did not involve data from other databases and did not involve a priori knowledge in our evaluation. Furthermore, the investigated population was relative small. Childhood acute lymphoblastic leukemia is a relative rare disease, with an incidence of 50–70 cases in a year in Hungary. In this respect the 543 ALL children in this study can be regarded as a large population. However, in a gene association study, where 62 SNPs are investigated, it is very difficult to detect in such a population weak effects or gene-gene interactions. Still, the BN-BMLA was able to reveal, besides the strongly relevant *ARID5B* and *IKZF1* polymorphisms, several possible interactions, and showed the possible types of them. According to our studies in larger, artificial datasets, if there are real complex interactions among the variables, the method is able to reveal complex network of interactions, significantly more complex than in Figure 1 in this study [12].

The Bayesian statistical framework allows the calculations of posteriors over a wide range of hypotheses, such as strong relevance of variables, pairs of variables, triplets of variables, etc. [9-13]. This shows the advantage of the Bayesian framework, because it allows the selection of appropriate level of complexity of hypotheses, which is not possible in the traditional hypothesis testing approach. Furthermore, this Bayesian global relevance analysis method provides posteriors, which are direct statements about hypotheses, thus it can also be used to construct probabilistic data analytic knowledge bases in genetic association studies to support complex quering, off-line meta-analysis, and fusion with background knowledge.

We offer the BN-BMLA method for academic purposes. The tool is available at a public website [38].

## Conclusion

In the present study we confirmed the role of genetic variations in *ARID5B* and *IKZF1* in the susceptibility to B-cell ALL. In our population we found that genetic variations in the *STAT3* gene might influence the susceptibility to hyperdiploid ALL. We presented the survival rate of a relatively large number of Hungarian children with ALL, and investigated the effects of some genetic and other variables on the survival rate. We also evaluated our results with our newly developed BN-BMLA method, which confirmed the relevance of SNPs in the *ARID5B* and *IKZF1* genes in B-cell ALL with high posterior probabilities. Furthermore, the results of the analysis gave additional information about the nature of the relations between several SNPs and the disease. In the different subgroups of patients, the BN-BMLA revealed several types of relations. Evaluating the survival rates of the patients, the BN-BMLA showed that besides risk groups and subtypes, genetic variations in the *BAX* and *CEBPA* genes might also influence the survival of the patients.

In the present paper we showed several advantageous features of the BN-BMLA method, and we demonstrated that in gene association studies it might be a useful supplementary to the traditional frequentist-based statistical method.

## Abbreviations

ALL: Acute lymphoblastic leukemia; BN-BMLA: Bayesian network based Bayesian multilevel analysis of relevance; DAG: Directed acyclic graph; EFS: Event-free survival; OS: Overall survival; HWE: Hardy-Weinberg Equilibrium; LD: Linkage Disequilibrium; BFM: Berlin-Frankfurt-Münster protocol; SNP: Single nucleotide polymorphism; FDR: False discovery rate; MC^3^: Metropolis Coupled Markov Chain Monte Carlo method.

## Competing interests

The authors of this paper declare that they have no competing interests.

## Authors’ contributions

OLC: Conceived and designed the experiments, participated in the drafting of the manuscript, evaluated the results; AG: Designed the software used in analysis, carried out Bayesian statistical evaluation, participated in the drafting of the manuscript; ÁFS: participated in the drafting of the manuscript, conceived and designed the experiments; DJE: participated in the drafting of the manuscript, was responsible for clinical data; PA: designed the software used in analysis, carried out statistical evaluation; GS: carried out the molecular genetic studies, evaluated the results; NK: carried out the molecular genetic studies; KC: was responsible for clinical data; MH: evaluated the results, was responsible for the clinical data; GK: was responsible for the clinical data; AF: participated in the drafting of the manuscript; CS: organized the study, participated in the design of the study and performed the statistical analysis, participated in the drafting of the manuscript. All authors read and approved the final manuscript.

## Pre-publication history

The pre-publication history for this paper can be accessed here:

http://www.biomedcentral.com/1755-8794/5/42/prepub

## Supplementary Material

Additional file 1 Minor allele and genotype frequencies (%) in pediatric ALL (n =543) and control (n = 529) patients. Click here for file

Additional file 2 Results of the statistical analysis of all genotyping data. Click here for file

Additional file 3 Linkage disequilibrium between some SNP pairs. Click here for file

Additional file 4 **Haplotypes in the *IKZF1 *gene, which significantly influenced the risk of ALL.** Click here for file

Additional file 5 Survival rates in the different genotype groups according to rs11667351 in the BAX gene. Click here for file

Additional file 6 Results of the statistical evaluations of the influence of some SNPs on the overall survival time. Click here for file

Additional file 7 The a posteriori probabilities of the different association types in ALL susceptibility. Click here for file

Additional file 8 The a posteriori probabilities of the different association types in event-free and overall survival. Click here for file
