# Supplementary figures and images for: Candidate gene association study in pediatric acute lymphoblastic leukemia evaluated by Bayesian network based Bayesian multilevel analysis of relevance
Source: BMC Med Genomics. 2012 Sep 28;5:42. doi: 10.1186/1755-8794-5-42 (PMC3542204; doi:10.1186/1755-8794-5-42)

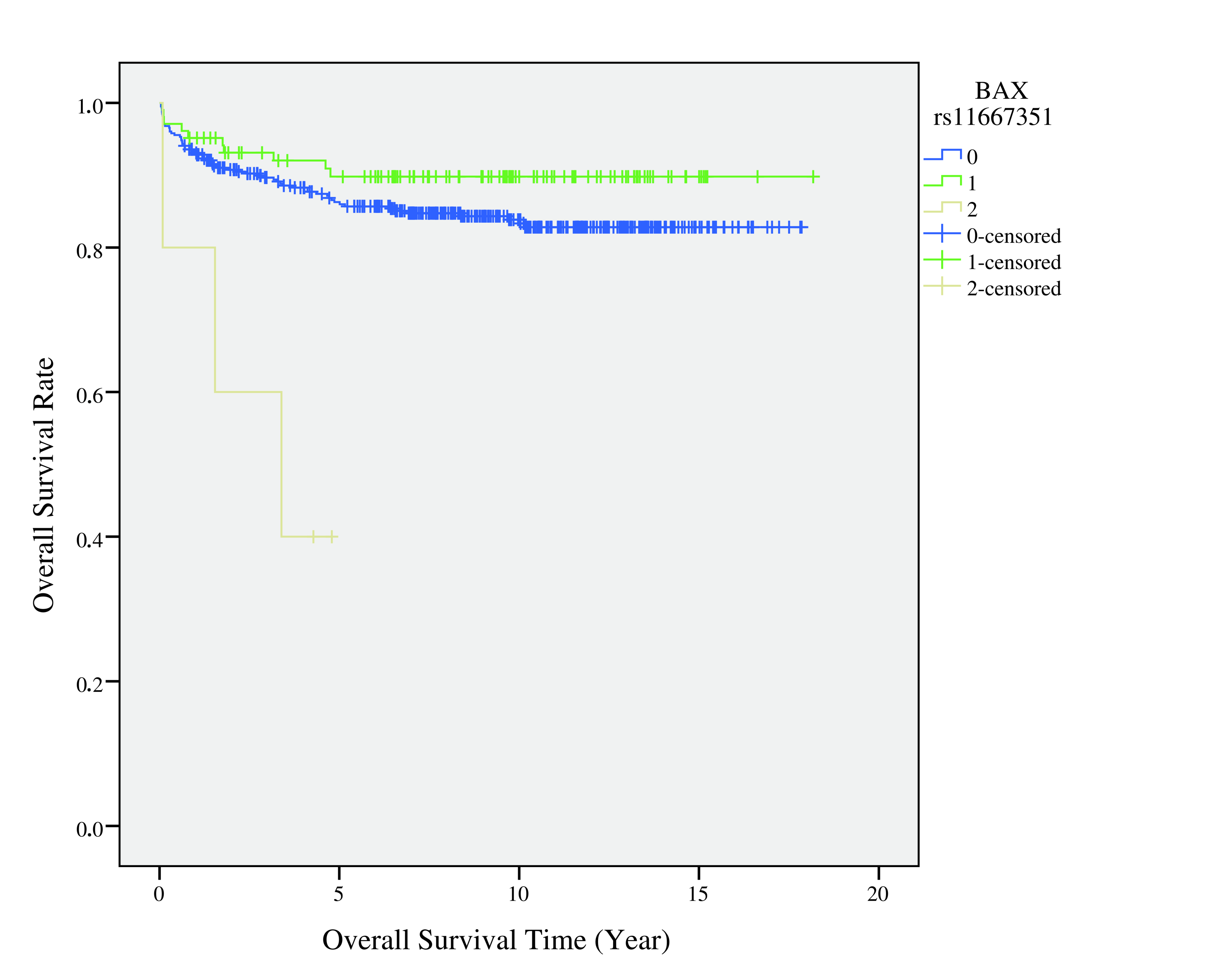

Supplement: Additional file 5 — Survival rates in the different genotype groups according to rs11667351 in the BAX gene. [file 1755-8794-5-42-S5.jpeg]
